# Supplementary material for: Resolving the full spectrum of human genome variation using Linked-Reads
Source: Genome Res. 2019 Apr;29(4):635–45. doi: 10.1101/gr.234443.118 (PMC6442396; doi:10.1101/gr.234443.118)
Supplement: Supplemental Material [file supp_gr.234443.118_Supplemental_longranger-2.2.zip › longranger-2.2.tar.bz2!/lr2.2-public/lib/stan/cmdstan/stan/doc/api/html/inverse__spd_8hpp_source.html]

Stan: src/stan/math/matrix/inverse\_spd.hpp Source File


|  |  |
| --- | --- |
|  | Stan  2.5.0  probability, sampling & optimization |


- Main Page
- Related Pages
- Namespaces
- Classes
- Files

- File List
- File Members

All Classes Namespaces Files Functions Variables Typedefs Enumerations Enumerator Friends Macros Pages

- src
- stan
- math
- matrix

inverse\_spd.hpp

Go to the documentation of this file.

1 #ifndef STAN\_\_MATH\_\_MATRIX\_\_INVERSE\_SPD\_HPP

2 #define STAN\_\_MATH\_\_MATRIX\_\_INVERSE\_SPD\_HPP

3

4 #include <stan/math/matrix/Eigen.hpp>

5 #include <stan/math/error\_handling/matrix/check\_square.hpp>

6 #include <stan/math/error\_handling/matrix/check\_symmetric.hpp>

7

8 namespace stan {

9  namespace math {

10

16  template <typename T>

17  inline

18  Eigen::Matrix<T,Eigen::Dynamic,Eigen::Dynamic>

19  inverse\_spd(const Eigen::Matrix<T,Eigen::Dynamic,Eigen::Dynamic>& m) {

20  stan::math::check\_square("inverse\_spd(%1%)",m,"m",(double\*)0);

21  stan::math::check\_symmetric("inverse\_spd(%1%)",m,"m",(double\*)0);

22  Eigen::LDLT< Eigen::Matrix<T,Eigen::Dynamic,Eigen::Dynamic> > ldlt(0.5\*(m+m.transpose()));

23  if (ldlt.info() != Eigen::Success)

24  throw std::domain\_error("Error in inverse\_spd, LDLT factorization failed");

25  if (!ldlt.isPositive() || (ldlt.vectorD().array() <= 0).any())

26  throw std::domain\_error("Error in inverse\_spd, matrix not positive definite");

27  return ldlt.solve(Eigen::Matrix<T,Eigen::Dynamic,Eigen::Dynamic>::Identity(m.rows(),m.cols()));

28  }

29

30  }

31 }

32 #endif

stan::math::inverse\_spd

Eigen::Matrix< T, Eigen::Dynamic, Eigen::Dynamic > inverse\_spd(const Eigen::Matrix< T, Eigen::Dynamic, Eigen::Dynamic > &m)

Returns the inverse of the specified symmetric, pos/neg-definite matrix.

**Definition:** inverse\_spd.hpp:19

check\_symmetric.hpp

stan::math::check\_symmetric

bool check\_symmetric(const char \*function, const Eigen::Matrix< T\_y, Eigen::Dynamic, Eigen::Dynamic > &y, const char \*name, T\_result \*result)

Return true if the specified matrix is symmetric.

**Definition:** check\_symmetric.hpp:31

Eigen.hpp

stan::math::check\_square

bool check\_square(const char \*function, const Eigen::Matrix< T\_y, Eigen::Dynamic, Eigen::Dynamic > &y, const char \*name, T\_result \*result)

Return true if the specified matrix is square.

**Definition:** check\_square.hpp:24

check\_square.hpp


---

[ Stan Home Page ]

*© 2011–2014,
Stan Development Team.*
